# Characterization and Mapping of Leaf Rust and Stripe Rust Resistance Loci in Hexaploid Wheat Lines UC1110 and PI610750 under Mexican Environments

**DOI:** 10.3389/fpls.2017.01450

**Published:** 2017-08-21

**Authors:** Caixia Lan, Iago L. Hale, Sybil A. Herrera-Foessel, Bhoja R. Basnet, Mandeep S. Randhawa, Julio Huerta-Espino, Jorge Dubcovsky, Ravi P. Singh

**Affiliations:** ^1^International Maize and Wheat Improvement Center Mexico City, Mexico; ^2^Department of Biological Sciences, University of New Hampshire, Durham NH, United States; ^3^Campo Experimental Valle de Mexico, Instituto Nacional de Investigaciones Forestales, Agrícolas y Pecuarias Chapingo, Mexico; ^4^Department of Plant Sciences, University of California, Davis, Davis CA, United States

**Keywords:** adult plant resistance, durable rust resistance, leaf rust, stripe rust, wheat

## Abstract

Growing resistant wheat varieties is a key method of minimizing the extent of yield losses caused by the globally important wheat leaf rust (LR) and stripe rust (YR) diseases. In this study, a population of 186 F_8_ recombinant inbred lines (RILs) derived from a cross between a synthetic wheat derivative (PI610750) and an adapted common wheat line (cv. “UC1110”) were phenotyped for LR and YR response at both seedling and adult plant stages over multiple seasons. Using a genetic linkage map consisting of single sequence repeats and diversity arrays technology markers, in combination with inclusive composite interval mapping analysis, we detected a new LR adult plant resistance (APR) locus, *QLr.cim-2DS*, contributed by UC1110. One co-located resistance locus to both rusts, *QLr.cim-3DC/QYr.cim-3DC*, and the known seedling resistance gene *Lr26* were also mapped. *QLr.cim-2DS* and *QLr.cim-3DC* showed a marginally significant interaction for LR resistance in the adult plant stage. In addition, two previously reported YR APR loci, *QYr.ucw-3BS* and *Yr48*, were found to exhibit stable performances in rust environments in both Mexico and the United States and showed a highly significant interaction in the field. *Yr48* was also observed to confer intermediate seedling resistance against Mexican YR races, thus suggesting it should be re-classified as an all-stage resistance gene. We also identified 5 and 2 RILs that possessed all detected YR and LR resistance loci, respectively. With the closely linked molecular markers reported here, these RILs could be used as donors for multiple resistance loci to both rusts in wheat breeding programs.

## Introduction

Wheat leaf rust (LR) and stripe rust (YR), caused by the air-borne fungi *Puccinia triticina* (*Pt*) and *Puccinia striiformis* f. sp. *tritici* (*Pst*), respectively, are considered the primary biotic threats to wheat production globally (Todorovska et al., 2009). LR can result in 20–40% yield losses resulting in dying leaves, reduced floret set, and grain shriveling (Knott and Padidam, 1988); and the potential yield loss for YR-susceptible cultivars was determined to be more than 60% in the Pacific Northwest (Chen, 2014). Recently, Hovmøller et al. (2015) found that the highly aggressive and heat-tolerant “Warrior” and “Kranich” races of YR originated from a sexually recombining population in the center of diversity of the YR fungus near the Himalayan region of central Asia. A new variant of the aggressive YR race group common in North America (Chen, 2005) caused a widespread epidemic in the highlands of Mexico in 2014 on wheat variety “Nana F2007” due to its combination of virulence on resistance genes *Yr3, Yr27*, and *Yr31* (Huerta-Espino et al., 2015). Breeding for durable resistance to wheat rusts continues to be a global priority but is also a challenge due to the complexity of interactions among resistance genes as well as the wide diversity and continuous evolution of pathogen races (Lowe et al., 2011a;Vazquez et al., 2015).

Genetic resistance to rust pathogens is commonly characterized as belonging to one of the following three general categories: (1) race-specific seedling resistance, also known as all-stage resistance (Chen, 2013); (2) race-specific adult plant resistance (APR); and (3) race non-specific APR, also known as slow-rusting or partial resistance (Johnson and Law, 1973; Das et al., 1992). Several all-stage, race-specific resistance genes characterized in wheat are known to be associated with various degrees of hypersensitive response (McIntosh et al., 2016) through elicitation of programmed cell death in the host if the pathogen possesses the corresponding avirulence genes (Flor, 1942). Some race-specific APR genes are known for both YR (e.g., *Yr11, Yr12, Yr13, Yr14, Yr16, Yr39*, and *Yr49*) and LR (e.g., *Lr13, Lr22a*, and *Lr22b*) (Seyfarth, 2000; McIntosh et al., 2016). These two race-specific resistances are frequently non-durable under high selection pressure on the pathogen due to rapid mutation rates and dependence on a direct protein–protein interaction for recognition (Jones and Dangl, 2006). In contrast, race non-specific APR genes generally do not provide a high level of resistance in isolation, but can do so when deployed in combination with other minor genes (Singh et al., 2000a). To date, more than 76 LR and 78 YR resistance genes have been cataloged (McIntosh et al., 2016). Of these, only *Lr34/Yr18/Pm38/Sr57* (Singh et al., 2012), *Lr46/Yr29/Pm39/Sr58* (Singh et al., 2013), and *Lr67/Yr46/Pm46/Sr55* (Herrera-Foessel et al., 2014) confer pleiotropic, race non-specific APR to LR, YR, powdery mildew, and stem rust. The molecular structures of *Lr34/Yr18* (Krattinger et al., 2009) and *Lr67/Yr46* (Moore et al., 2015) indicate different underlying mechanisms of the transporter-mediated APR conferred by these genes.

Over the past 20 years, more than 50 quantitative trait loci (QTL) for LR and YR resistance have been reported using a wide range of markers, including diversity arrays technology (DArT), single sequence repeats (SSRs), and single-nucleotide polymorphisms (Rosewarne et al., 2013; Li et al., 2014; Maccaferri et al., 2015); and the genetic locations of these QTL are continually being refined through subsequent mapping studies. In a previous study, Lowe et al. (2011b) reported moderate levels of field resistance to YR in both the California (CA)-adapted hexaploid wheat breeding line UC1110 and the synthetic wheat derivative PI610750. Using a population of 186 recombinant inbred lines (RILs) derived from a cross between these parents, four YR resistance QTL (two from UC1110 and two from PI610750) were identified and characterized under the CA rust environment. The objectives of this follow-up study were to: (1) investigate the performance of these previously reported YR resistance loci in the Mexican rust environment; (2) identify potential sources of both seedling resistance and race non-specific APR to both LR and YR in this mapping population; and (3) Determine interactions between the identified resistance loci in the adult plant stage.

## Materials and Methods

### Plant Materials

A population of 186 F_7_-derived F_8_ RILs was developed from a cross between UC1110 (pedigree: Chukar/3/Yding//Bluebird/Chanate) and PI610750 [pedigree: Croc1/*Aegilops tauschii* (Synthetic 205)//Kauz] (Lowe et al., 2011b). UC1110, an adapted Californian hard white spring wheat line bred by and used as a parent in the University of California, Davis (UC Davis) wheat breeding program, carries YR resistance loci on wheat chromosomes 2BS and 3BS (Lowe et al., 2011b) under the CA rust environment. PI610750, a synthetic derivative spring wheat line developed by CIMMYT in Mexico, carries the YR APR gene *Yr48* and a minor YR resistance locus on chromosome 2AS (Lowe et al., 2011b). After multiplication of the RILs and parents in Mexico, F_8_ seeds were used for all genotyping and disease phenotyping.

### Greenhouse Experiments (Seedling Stage)

#### Leaf Rust

The two parents and 186 RILs were tested in January 2014 under greenhouse conditions with *P. triticina* race MBJ/SP [avirulence/virulence formula: *Lr2a, 2b, 2c, 3ka, 9, 16, 18, 19, 21, 24, 25*, (*26*), *28, 29, 30, 32, 33, 36/1, 3, 3bg, 10, 11, 13, 15, 17, 20, 23, 27+31* (Lan et al., 2014b)]. A set of 48 differential lines with known LR resistance genes (most in the Thatcher background, including Thatcher+*Lr26*) were included in the seedling tests to compare the infection types (ITs) of the parents and RILs to those associated with known resistance genes. All RILs were sown in trays in hills of six to eight seeds each (48 lines/tray). Once seedlings reached the two-leaf stage, they were inoculated by spraying urediniospores suspended in lightweight Soltrol 170 mineral oil using an atomizer. To facilitate evaporation of the oil, the inoculated plants were left on an open bench for 20 min before being transferred into an 18°C dew chamber overnight, then back to the greenhouse. Based on a monitor that measured air temperature in the greenhouse every 30 min, the minimum, maximum, and mean post-inoculation temperatures were 10.2, 25.8, and 20.0°C, respectively. LR ITs were recorded 11 days post-inoculation based on the following scale modified from Roelfs et al. (1992): “0” = no visible symptoms; “;” = only necrotic/chlorotic flecks without any uredinia; “1” = small uredinia surrounded by necrosis; “2” = small to medium uredinia surrounded by chlorosis or necrosis; “3” = medium-sized uredinia without chlorosis or necrosis; “4” = large-sized uredinia without chlorosis or necrosis; “X” = random distribution of variable-sized uredinia; “3C3” = medium-sized uredinia with chlorosis; “;11+” = necrotic/chlorotic flecks with small to medium uredinia; and “+” and “-” were used when uredinia were somewhat larger or smaller than normal for the ITs. ITs of 3, 3+, and 4 are considered to be susceptible host reactions, whereas all of other ITs are considered resistant.

#### Stripe Rust

In January of 2016 and 2017, the parents and RILs were evaluated for their reaction to YR at the seedling stage using the recent Mexican *P. striiformis* isolate Mex14.191 virulent on *Yr* genes *2, 3, 6, 7, 8, 9, 17, 27, 31*, and *A* (Huerta-Espino et al., 2015). A total of 30 differential lines with known YR resistance genes (mostly in Avocet background) were also included. The inoculation method was the same as described above for LR, but with a lower incubation temperature in the dew chamber of 7°C. Inoculated plants were placed in the dew chamber for 24 h and then transferred to the greenhouse. The minimum, maximum, and mean post-inoculation greenhouse temperatures were 7.4, 27.5, and 14.9°C, respectively. ITs were recorded approximately 2 weeks post-inoculation, based on a 0–9 scale modified from McNeal et al. (1971), where “0” = no visible infection; “1” = necrotic/chlorotic flecks without sporulation; “2” = necrotic/chlorotic stripes without sporulation; “3” = necrotic/chlorotic stripes with trace sporulation; “4” = necrotic/chlorotic stripes with light sporulation; “5” = necrotic/chlorotic stripes with intermediate sporulation; “56” = necrotic/chlorotic stripes with intermediate to moderate sporulation; “6” = chlorotic stripes with moderate sporulation; “7” = stripes without chlorosis or necrosis and with moderate sporulation; “8” = stripes without chlorosis or necrosis and with sufficient sporulation; and “9” = stripes without chlorosis or necrosis and abundant sporulation. ITs of 0–4, 5–6, and 7–9 are categorized as resistant, intermediate, and susceptible, respectively.

### Field Experiments (Adult Plant Stage)

The parents and RIL population were evaluated for APR to LR at Ciudad Obregón in the Yaqui Valley in northern Mexico during the 2011–2012 growing season (LR2012Y) and at El Batán, CIMMYT headquarters outside Mexico City, in the 2012 and 2013 seasons (LR2012B and LR2013B). Similarly, two YR experiments were conducted at another CIMMYT research station in Toluca, Mexico, during the 2012 and 2013 growing seasons (YR2012 and YR2013). Field plots consisted of 0.7-m paired rows planted with approximately 60 plants of each line. The Avocet near-isoline for *Yr24/26* was used as the LR spreader, whereas a mixture of susceptible wheat lines comprised of cv. “Morocco,” an Avocet near-isoline for gene *Yr31*, and six lines derived from an Avocet/Attila cross, was used as the YR spreader in field trials. The spreaders were planted around the experimental area and as hill plots in the middle of a 0.3-m pathway on one side of each experimental plot. The LR epidemic was initiated by spraying the LR spreader with an equal mixture of *P. triticina* races MBJ/SP and MCJ/SP suspended in Soltrol 170. The main difference between the two races is partial (MBJ/SP) and complete (MCJ/SP) virulence on *Lr26* (Lan et al., 2014b). For the YR trials, a mixture of *P. striiformis* races Mex96.11 and Mex08.13 was sprayed onto the spreaders within and around the experimental areas. Isolate Mex08.13 belongs to the lineage of the aggressive YR race that was first detected in California (Chen, 2005) and is virulent on *Yr* genes *2, 6, 7, 8, 9, 31*, and *A*, whereas Mex96.11 is virulent on *Yr* genes *2, 3, 6, 7, 9, 27*, and *A* (Lan et al., 2014b). Disease severities (DS) for the parents and the RILs were recorded three times in each experiment, according to the modified Cobb’s scale (Peterson et al., 1948) and host response to infection was determined according to Roelfs et al. (1992), where “R” = resistant, or miniature uredinia surrounded by necrotic tissues; “MR” = moderately resistant, or smaller to moderate-sized uredinia surrounded by necrotic or chlorotic tissues; “MS” = moderately susceptible, or moderate-sized uredinia without necrotic or chlorotic tissues; and “S” = susceptible, or large uredinia without necrotic or chlorotic tissues. In cases of repeated DS data, the first note was recorded when the susceptible check Avocet displayed approximately 80% severity and repeated about a week later when it reached 90–100%. For multiple disease readings, the area under the disease progress curve (AUDPC) was calculated using the method described by Bjarko and Line (1988). Final DS (FDS) and AUDPC scores were used in downstream genetic analyses.

### Molecular Markers, Linkage Map Construction, and QTL Analysis

In this study, baseline molecular marker data and linkage map information for the UC1110/PI610750 population was used from a previous study by Lowe et al. (2011b). Linkage maps were graphically produced by using MapChart (Voorrips, 2002). QTL mapping of FDS and AUDPC for each experiment was carried out using inclusive composite interval mapping (ICIM) 4.1 (Meng et al., 2015) The ICIM method was also used to analyze the final mean DS for each disease (LRM and YRM) across experiments. The logarithm of odds (LOD) threshold to declare a QTL significant at *P* = 0.05 for each trait was determined based on 1,000 permutation tests. Stepwise regression was used to estimate both the main effects and percentages of phenotypic variance explained (PVE) by individual QTL (at LOD peaks). The QTL result was confirmed by QTL Cartographer (Wang et al., 2012). The seedling resistance gene was mapped by the Joinmap 4.1 software (van Ooijen, 2006). Marker orders and chromosomal assignments of linkage groups were verified using wheat SSR consensus genetic maps and the physical bin locations of DArT markers (Somers et al., 2004; Francki et al., 2009; Huang et al., 2012; Wilkinson et al., 2012). QTL designations were assigned following recommended practices^1^. Factorial analyses of variance (ANOVA) were conducted to test for the significances of interactions between detected resistance loci based on AUDPC.

## Results

### Seedling Responses to LR and YR

#### Leaf Rust

Seedling ITs were “;11+” and “3C3” for P1610750 and UC1110, respectively, against *P. triticina* race MBJ/SP (data not shown). The seedling resistance contributed by PI610750 was mapped to wheat chromosome 1B, 23.2 cM proximal of a molecular marker cluster comprised of two SSR and 173 DArT markers (Supplementary Table S1). This line displayed a similar IT to the *Lr26* tester (1RS.1BL translocation), indicating that *Lr26* may be the gene conferring seedling resistance to LR race MBJ/SP in PI610750. This is also supported by the presence of the 1RS.1BL translocation in PI610750, its source most likely being one of its parents, cv. “Kauz” (Lowe et al., 2011b).

#### Stripe Rust

**FIGURE 1:**
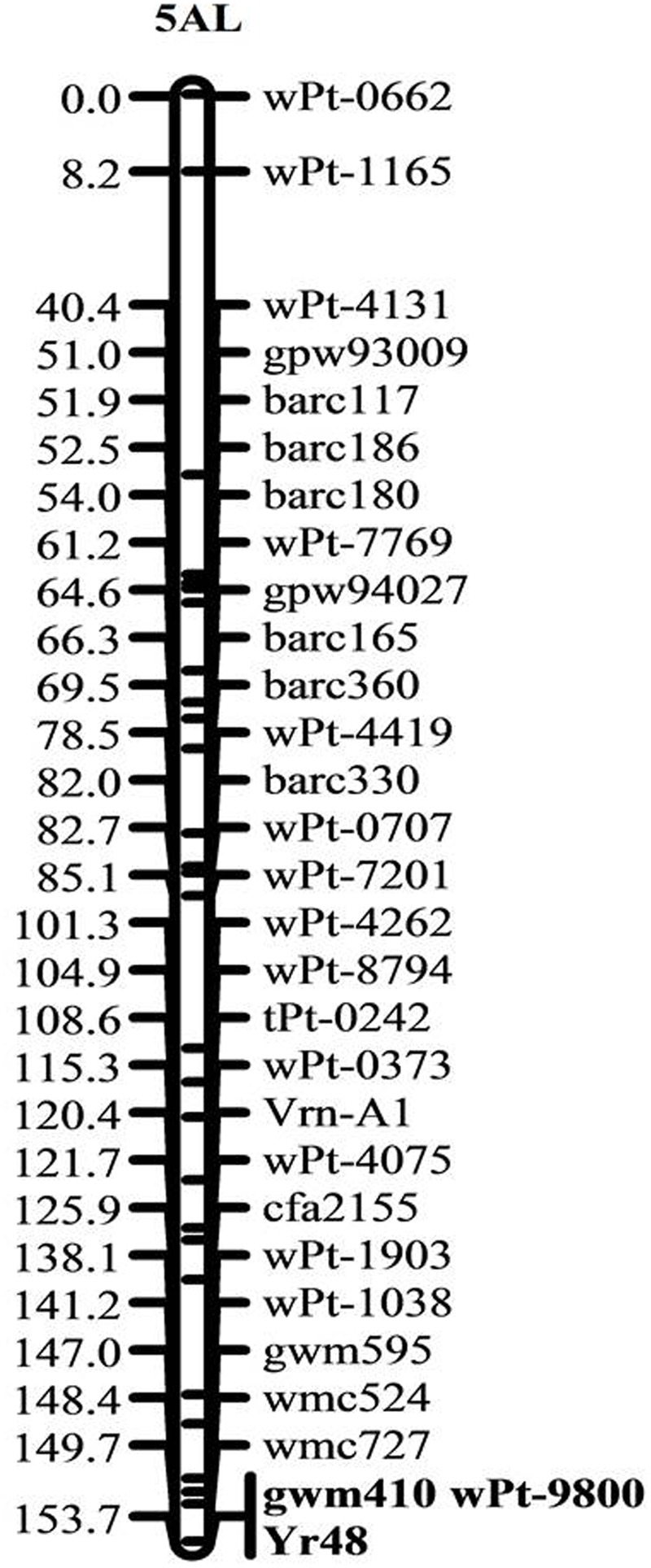
Genetic linkage map of chromosome 5A showing the location of the all-stage resistance gene *Yr48*. Locus names and corresponding locations on the genetic map are indicated on the right side. Map distances in cM are shown on the left side. Markers co-segregating with *Yr48* are given in bold.

**FIGURE 2:**
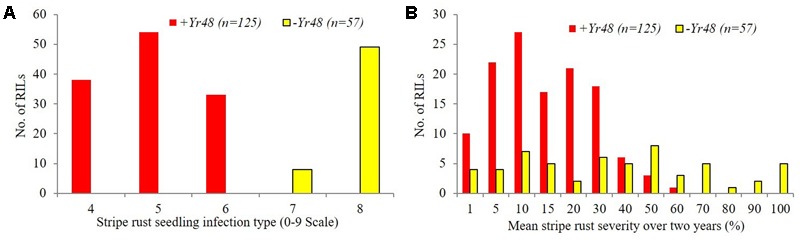
Frequency distributions of stripe rust response for UC1110/PI610750 recombinant inbred lines (RILs) possessing and lacking *Yr48* at the seedling stage **(A)** and the adult plant stage **(B)**.

The ITs of PI610750 and UC1110 were “56” and “6,” respectively, against *P. striiformis* isolate Mex14.191 at the seedling stage. The seedling resistance gene from PI610750 was mapped to the proximal end of chromosome 5AL, where it co-segregated with molecular markers *gwm410* and *wPt-9800* (**Figure 1**). This chromosome position coincides with the location of *Yr48* reported in the previous study by Lowe et al. (2011b), thus indicating that *Yr48* confers an intermediate level of seedling resistance to race Mex14.191. The seedling YR IT ranged from 4 to 6 for RILs carrying *Yr48*, whereas it was 7 to 8 for RILs lacking this resistance gene (**Figure 2A**). Mean YR DS ranged from 1 to 60% for RILs carrying *Yr48*, whereas it was 1 to 100% for RILs without it (**Figure 2B**). Overall, 125 RILs exhibited ITs ranging from 4 to 6 (resistant), while 57 RILs exhibited ITs ranging from 7 to 8 (susceptible), a result which indicates a distorted distribution for this gene in the population (*P* < 0.0001). Similar distortion (i.e., 122–129 RILs carrying the PI610750 allele and 57–63 RILs carrying the UC1110 allele (*P* < 0.0001) was observed for the five molecular markers most closely linked to *Yr48* (Supplementary Table S2).

### Adult Plant Responses to LR and YR

**FIGURE 3:**
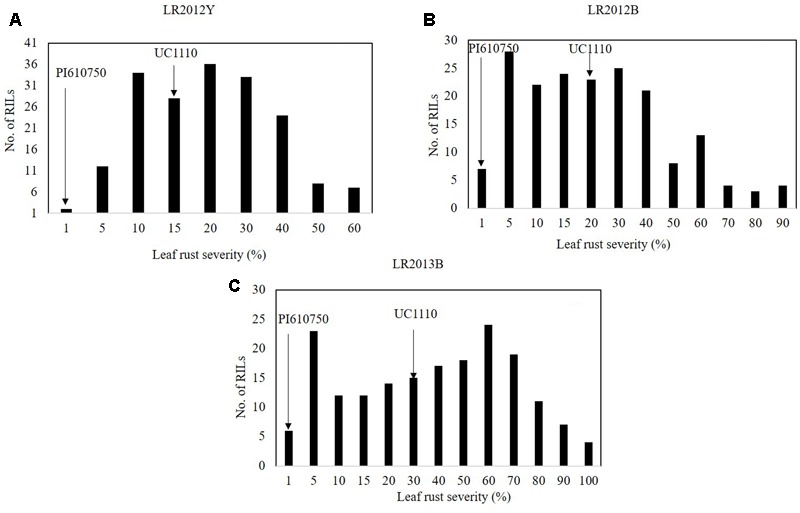
Frequency distributions of UC1110/PI610750 recombinant inbred lines (RILs) for final leaf rust severity in field trials **(A)** at Ciudad Obregón in 2012 (LR2012Y, **(B)** at El Batán in 2012 (LR2011B), and **(C)** at El Batán in 2013 (LR2012B). The leaf rust severities for PI610750 and UC1110 are indicated by arrows.

The FDS for LR (LRM) were 1 MR for PI610750 and 15 MSS, 20 MSS, and 30 MSS for UC1110 in LR2012Y, LR2012B, and LR2013B, respectively, over the three seasons of this study (**Figures 3A–C**); and LRM for the RILs ranged from 18 to 41% in those same environments (data not shown). LR severities among the RILs was continually distributed across 3 years (**Figure 3**), indicating quantitative inheritance of APR to LR in the population.

**FIGURE 4:**
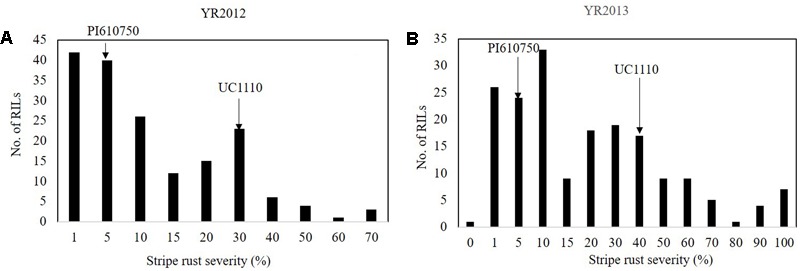
Frequency distributions of UC1110/PI610750 recombinant inbred lines (RILs) for final stripe rust severity in field trials **(A)** at Toluca in 2012 (YR2012) and **(B)** at Toluca in 2013 (YR2013). The stripe rust severities for PI610750 and UC1110 are indicated by arrows.

For YR, the FDS (YRMs) for PI610750 were 5 MR and for UC1110 were 30 M and 40 M in YR2012 and YR2013, respectively (**Figures 4A,B**). YRM for the RILs ranged between 14 and 30% across the two seasons (**Figure 4**). The frequency distributions of YR severity among the RILs showed a bimodal distribution in both environments (**Figure 4**), which is an indication for the segregation of a major seedling resistance gene in the UC1110/PI610750 population.

Pearson correlation coefficients (*r*) of LR severity ranged from 0.57 to 0.81 among the three environments, whereas it was 0.66 for YR severity over the 2 years (**Table 1**). Low and non-significant correlations were observed between LR and YR severities (*r* = 0.03–0.25) across all environments.

**Table 1:** Pearson’s correlation coefficients (*r*) among the UC1110/PI610750 RILs for final leaf rust severities in three environments (Ciudad Obregón LR2012Y, El Batán LR2012B and LR2013B) and stripe rust severities in two (Toluca YR2012 and YR2013) environments in Mexico.

Environment	LR2012Y	LR2012B	LR2013B	YR2012
LR2012B	0.57^∗∗^			
LR2013B	0.58^∗∗^	0.81^∗∗^		
YR2012	0.24	0.18	0.25	
YR2013	0.23	0.03	0.14	0.66^∗∗^

*^∗∗^*P* < 0.0001.*

### APR Locus to LR on Chromosome 2DS

**FIGURE 5:**
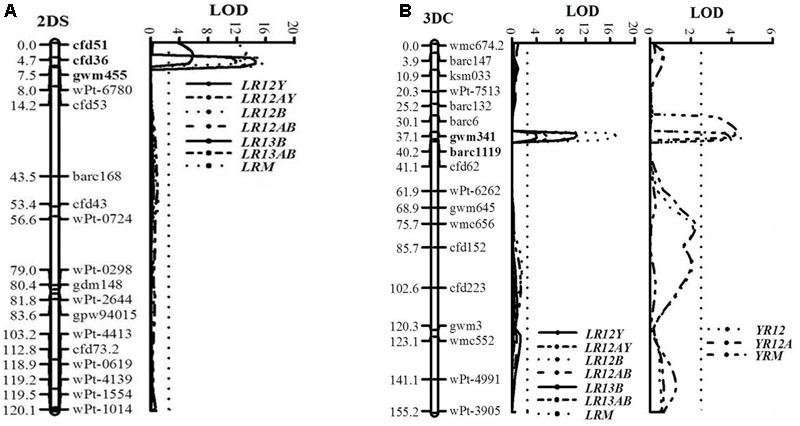
LOD plots of quantitative trait loci (QTL) for adult plant resistance to leaf rust on chromosomes 2DS **(A)** and to both leaf and stripe rust on chromosome 3DC **(B)**, identified using IciMapping 4.1 in the UC1110/PI610750 RIL population. Significant LOD were detected based on 1,000 permutations. Positions (cM) of the molecular markers on chromosomes are shown on the vertical axes; cumulative genetic distances of linkage groups are also shown. LR12Y, LR12B, and LR13B: leaf rust phenotypic data at Ciudad Obregón in 2012 and El Batán during 2012 and 2013, respectively; YR12: stripe rust phenotypic data at Toluca in 2012; LR12AY, LR12AB, LR13AB, and YR12A: area under the disease progress curve (AUDPC); LRM and YRM: mean final disease severity over test environments. QTL flanking markers are in bold.

**FIGURE 6:**
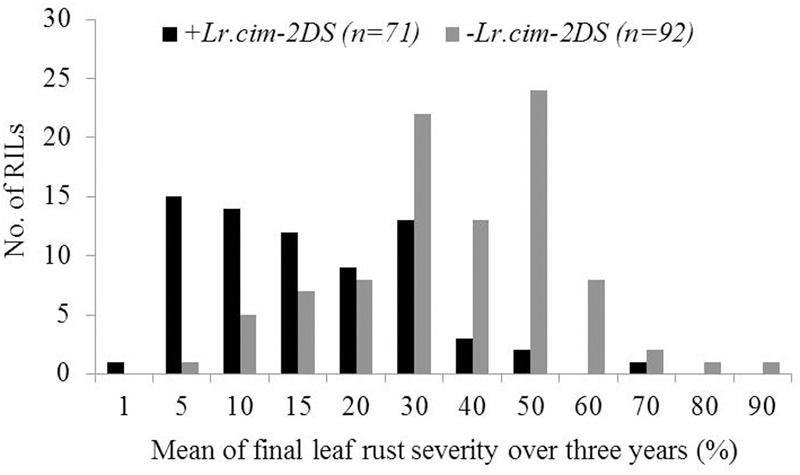
Comparison of UC1110/PI610750 recombinant inbred lines (RILs) for mean final leaf rust severity in the presence (+) and absence (–) of the leaf rust resistance QTL *Lr.cim-2DS* over 3 years of field trials. The numbers of RILs in each category are shown in parentheses.

A major QTL for LR resistance, *QLr.cim-2DS*, was located on the end of the short arm of chromosome 2D and interval flanked by SSR markers *cfd51* and *gwm455* (**Figure 5A**). This QTL was consistently detected in all three LR field experiments and explained 11.8–26.6% of the observed variation in LRM (**Table 2**). Mean LR severities for RILs carrying *QLr.cim-2DS* ranged from 1 to 50% (with an exception of entry 86 with 70% LR FDS), whereas it was 5 to 90% for RILs without this locus (**Figure 6**). This resistance locus derives from UC1110 (**Table 2**).

**Table 2:** Quantitative trait loci (QTL) detected in the UC1110/PI610750 RIL population for adult plant resistance to leaf rust (Ciudad Obregón LR2012Y, El Batán LR2012B and LR2013B) and stripe rust (Toluca YR2012 and YR2013); traits include the area under the disease progress curve (AUDPC, viz. LR2012AY) and the mean of final disease severity over all environment (LRM and YRM).

QTL/gene^a^	Trait name	Position	Left marker	Right marker	LOD	PVE (%)	Add	Resistance source
*QLr.cim-2DS*	LR2012Y	4	cfd51	cfd36	5.9	12.1	-4.4	UC1110
	LR2012AY	4	cfd51	cfd36	5.7	11.8	-49.1	UC1110
	LR2012B	3	cfd51	cfd36	13.4	26.5	-11.1	UC1110
	LR2012AB	7	cfd36	gwm455	13.4	26.6	-109.6	UC1110
	LR2013B	7	cfd36	gwm455	14.6	25.4	-14.1	UC1110
	LR2013AB	6	cfd36	gwm455	11.9	22.9	-113.9	UC1110
	LRM	7	cfd36	gwm455	15.5	20.8	-8.6	UC1110
*QLr.cim-3DC*	LR2012Y	40	gwm341	barc1119	4.0	8.2	-4.1	UC1110
	LR2012AY	40	gwm341	barc1119	4.1	7.7	-40.1	UC1110
	LR2012B	40	gwm341	barc1119	6.0	11.1	-5.8	UC1110
	LR2012AB	40	gwm341	barc1119	5.8	10.4	-68.4	UC1110
	LR2013B	39	gwm341	barc1119	10.6	17.8	-11.8	UC1110
	LR2013AB	39	gwm341	barc1119	9.2	17.2	-99.1	UC1110
	LRM	39	gwm341	barc1119	16.9	23.3	-9.2	UC1110
*QYr.cim-3DC*	YR2012M	40	gwm341	barc1119	4.5	8.9	-4.6	UC1110
	YR2012AM	40	gwm341	barc1119	3.9	7.8	-28.4	UC1110
	YRM	38	gwm341	barc1119	3.9	6.1	-4.2	UC1110
*QYr.cim-3BS*	YR2012M	6	cfb3417	barc133	7.4	12.2	-6.2	UC1110
	YR2012AM	6	cfb3417	barc133	4.9	9.8	-32.0	UC1110
	YR2013M	6	cfb3417	barc133	24.8	38.1	-13.0	UC1110
	YR2013AM	6	cfb3417	barc133	25.6	38.2	-101.8	UC1110
	YRM	6	cfb3417	barc133	25.4	33.8	-13.0	UC1110
*Yr48*-5AL	YR2012M	183	wmc727	gwm410	13.2	24.7	9.4	PI610750
	YR2012AM	183	wmc727	gwm410	7.0	15.2	44.2	PI610750
	YR2013M	184	gwm410	wPt-9800	17.2	25.1	14.1	PI610750
	YR2013AM	183	wmc727	gwm410	17.3	23.4	84.5	PI610750
	YRM	184	gwm410	wPt-9800	20.0	24.6	11.8	PI610750

*^a^QTL/genes that extend across single one-log support confidence intervals were assigned the same symbol.*

### Co-located APR Loci to LR and YR on Chromosome 3D

Co-located APR loci to both rusts, *QLr.cim-3DC/QYr.cim-3DC*, were mapped near the centromeric region of chromosome 3D (3DC), flanked by SSR markers *gwm341* and *barc1119* (**Table 2** and **Figure 5B**). This co-located resistance locus was also contributed by UC1110 and explained 23.3 and 6.1% of the observed variation in YRM and LRM, respectively (**Table 2**). In addition, the previous study reported YR resistance loci on chromosome 3BS and *Yr48* were also identified in the present study under Mexican rust environments (**Table 2**).

### Interactions between Resistance Loci for LR and YR

The F_8_ RILs were divided into groups based on the marker-inferred factorial combinations of the two LR resistance QTL and three YR resistance QTL (**Tables 3, 4**). Specifically, the flanking molecular markers for each QTL were used to infer the presence of parental alleles in each RIL. A marginally significant interaction (*P* = 0.037) between *QLr.cim-2DS* and *QLr.cim-3DC* on LR was observed across 3 years based on AUDPC (**Table 3**), which only explained 1.4% of LR AUDPC variation. *QYr.ucw-3BS* did not show a big effect on YR resistance at the adult plant stage when it was present alone in the adult plant stage (2.8% of the variation), but it showed a highly significant interaction with *Yr48* that explained 37.9% of the variation (**Table 4**). By contrast, no significant gene interaction between *QYr.ucw-3BS* × *QYr.cim-3DC, QYr.cim-3DC* × *Yr48*, and *QYr.cuw-3BS*×*Yr48*×*QYr.cim-3DC* was detected, indicating additive effects was present between them. RILs combing all of three YR resistance QTL displayed severity from 0.1 to 3.6% with a near immune response to YR.

**Table 3:** Two-way factorial analysis of variance (ANOVA) of area under disease progress curve (AUDPC) for leaf rust resistance loci, using years as blocks.

Source	No. of RILs	DF	Type III SS	Mean square	*F*-value	*Pr* > *F*	Variation (%)
YEAR		2	615360.1	307680.0	10.77	<0.0001	3.1
2DS	47	1	3281283.7	3281283.7	114.81	<0.0001	26.1
3DC	38	1	2278573.7	2278573.7	79.73	<0.0001	17.0
2DS×3DC	33	1	124795.7	124795.7	4.37	0.0372	1.4

**Table 4:** Three-way factorial analysis of variance (ANOVA) of area under disease progress curve (AUDPC) for stripe rust resistance loci, using years as blocks.

Source	No. of RILs	DF	Type III SS	Mean square	*F*-value	*Pr* > *F*	Variation (%)
YEAR		1	242019.0	242019.0	28.05	<0.0001	0.1
3BS	16	1	1990404.0	1990404.0	230.73	<0.0001	2.8
5AL	11	1	1158229.5	1158229.5	134.26	<0.0001	16.6
3DC	50	1	211609.8	211609.8	24.53	<0.0001	4.6
3BS × 5AL	19	1	727184.1	727184.1	84.3	<0.0001	37.9
3BS × 3DC	10	1	0.1	0.1	0	0.998	0
5AL × 3DC	28	1	286.7	286.7	0.03	0.8555	0
3BS × 5AL × 3DC	24	1	11503.6	11503.6	1.33	0.249	0.3

## Discussion

UC1110 showed a moderate level of APR to LR and YR under Mexican rust environments, whereas PI610750 remained highly resistant to both rusts at the adult plant stage and showed an intermediate reaction to YR at the seedling stage. Resistance to LR and YR in the UC1110/PI610750 population appears to be controlled by sources of seedling resistance as well as by APR loci. In a previous study, YR resistance in this population was attributed to four APR QTL, including *QYr.ucw-2BS* and *QYr.ucw-3BS* derived from UC1110 and *QYr.ucw-2AS* and *Yr48* contributed by PI610750 (Lowe et al., 2011b).

### Leaf Rust Seedling Resistance Gene Lr26

*Lr26* is known to be present in the wheat-rye 1RS.1BL chromosome translocation (McIntosh et al., 1995), and this translocation was widely used in wheat breeding programs worldwide during the 1980s and 1990s. For instance, 54% of the wheat cultivars grown in Bulgaria carry the 1RS.1BL translocation (Landjeva et al., 2006), approximately 38% of wheat cultivars released since 1980 contain this translocation in China (Zhou et al., 2004), and nearly 50% of CIMMYT’s around 2,000 advanced bread wheat breeding lines were homozygous for the 1RS.1BL translocation in 1988 (Villareal et al., 1991). However, its frequency has decreased to below 5% in current breeding materials due to the breakdown of resistance genes located on it and to poor quality associated with the translocation. The widespread deployment of the 1RS.1BL translocation was due to the fact that the 1RS arm carries resistance genes to multiple diseases (e.g., *Sr31, Yr9*, and *Pm8*) and positively affects various agronomic traits (Tang et al., 2008). Unfortunately, races fully virulent on *Lr26* were detected in most wheat-growing areas, including Europe, the Indian subcontinent, North America, and South Africa in last middle of 1990s (McIntosh et al., 1995), likely due to the widespread use of a single, major resistance gene over a large area. In the current study, Mexican LR race MBJ/SP was found to be partially virulent on *Lr26*, whereas another field race (MCJ/SP) was completely virulent on this gene. Thus, this study was not designed to detect the effect of *Lr26*. This gene is closely linked to the YR resistance gene *Yr9* (Mago et al., 2005); however, we did not detect the effect of *Yr9* because both races used in the field trials were virulent. *Lr26* should be contributed by one of PI610750’s parents, Kauz (pedigree: Jupateco F73/Bluejay//Ures T81; released in Mexico as Bacanora T88), carrying the 1RS.1BL translocation (Singh and Rajaram, 1991).

### Leaf Rust Resistance Locus on 2DS

The APR QTL for LR, *QLr.cim-2DS*, contributed by UC1110, is located on the short arm of chromosome 2D, in the 2DS-5 region of the DArT physical map (Francki et al., 2009) and flanked by SSR markers *cfd36* and *cfd51*. So far, five cataloged seedling resistance genes have been detected on the short arm of chromosome 2D, including *Lr2* (Ausemus et al., 1946), *Lr15* (McIntosh and Baker, 1968), *Lr22* (Hiebert et al., 2007), *Lr39* (Raupp et al., 2001), and *Lr41* (Sun et al., 2009). *Lr22* and *Lr39* were transferred from *A. tauschii*, whereas *Lr41* was introgressed from *Aegilops cylindrica* to common wheat. UC1110 (pedigree: Chukar///Yding//Bluebird/Chanate) is an adapted breeding line developed at UC Davis with no indications of these introgressions in its pedigree; therefore, *QLr.cim-2DS* is expected to be different from these three seedling genes. *Lr15* was mapped in the common wheat line “Kenya W1483”; however, the LR races MBJ/SP and MCJ/SP are virulent on this gene. Although the two races are avirulent to *Lr2*, the seedling test suggests that *QLr.cim-2DS* is not *Lr2*, as the ITs of both parents were “1” when challenged with the *Lr2*-virulent race TCT/QB at the seedling stage. Although several other LR resistance QTL have been mapped in wheat lines, Avocet (Rosewarne et al., 2012), CI 13227 (Xu et al., 2005), and Saar (Zhang et al., 2009) on the distal region of chromosome 2DS. They are located more than 10 cM away from *QLr.cim-2DS* based on the wheat consensus map (Somers et al., 2004). Thus, it is likely that *QLr.cim-2DS* is a previously undetected APR locus for LR in common wheat. A single locus mapping population is currently under development to fine map this resistance locus.

### Co-located Resistance QTL to both Rusts on 3DC

The pleiotropic resistance QTL, *QLr.cim-3DC/QYr.cim-3DC*, was detected near the centromere of chromosome 3D and flanked by SSR markers *gwm341* and *barc1119*. Basnet et al. (2014) detected *QLr.tam-3D/QYr.tam-3D* in the CIMMYT wheat line Quaiu#3 and assigned its location to a proximal region of chromosome arm 3DS, flanked by markers *wPt-672034* and *barc125*. That QTL was found to explain 3–6 and 3.2–4.3% of the observed variation in LR and YR DS, respectively. The flanking markers for the pleiotropic QTL in this study are only ∼4 cM distal of the *QLr.tam-3D/QYr.tam-3D locus*, based on the wheat consensus map (Somers et al., 2004). *QLr.cim-3DC* was also detected in an Avocet/Francolin#1 population (Lan et al., 2014a), where it explained 17.8–25.4% of the observed variation in LR DS in the *Lr46*-subtracted sub-population of RILs; but its effect was not significantly detectable on YR. In addition, the pedigree of UC1110 is Chukar/3/Yding//Bluebird/Chanate, while it is Babax/Lr42//Babax^∗^2/3/Vivitsi for Quaiu#3 (Basnet et al., 2014) and Waxwing^∗^2/Vivitsi for Francolin#1 (Lan et al., 2014b). The pedigree of Vivitsi is Sonoita F 81/Yaco/5/Bobwhite/Crow//Buckbuck/Pavon F 76/3/Yecora F 70/4/Trap#1, whereas Bluebird is one of parents for Buckbuck. Therefore, *QLr.cim-3DC/QYr.cim-3DC* in UC1110 might be the same QTL as in Quaiu#3 and Francolin#1 and derived from Bluebird, though a larger effect was observed in UC1110 than in either the Quaiu#3 or Francolin genetic backgrounds. Allelism tests will be required to test these hypotheses.

### Stripe Rust Resistance Loci

One YR APR QTL was mapped in this study to chromosome arm 3BS, within the marker interval *cfd3417*-*barc133*, in agreement with the previous study conducted under the California YR environment (Lowe et al., 2011b). In the previous study, the QTL was named *QYr.ucw-3BS* and was found to be closely linked to *gwm533.1, barc133*, and *wPt-1620*. An APR YR resistance gene, *Yr30*, has also been mapped on the short arm of chromosome 3B (Singh et al., 2000b); however, *QYr.ucw-3BS* is not *Yr30* based on allelism test (Lowe et al., 2011b). While this resistance locus did not offer any significant protection against US YR race PST-100, it did impart partial resistance at the adult plant stage. The IT for UC1110 was “6” against Mexican race Mex14.191, indicating this line might present a minor resistance gene with moderate-susceptible reaction at seedling stage.

The second YR resistance gene, *Yr48*, was mapped at the same genomic position as reported in the previous study (Lowe et al., 2011b). This resistance gene comes from the synthetic wheat derivative PI610750 and is located in the telomeric region of the long arm of chromosome 5A. *Yr48* showed large effect on YR in the adult plant stage under the U.S. rust environment, with an average reduction of 63% YR severity compared to fully susceptible lines (Lowe et al., 2011b), whereas it reduced YR severity only up to 37.1% under the Mexican rust environment. The ITs of RILs with *Yr48* ranged from four to six at seedling stage and field severity ranged from 1 to 60%. We conclude that *Yr48* confers moderate seedling resistance and APR against the Mexican *Pst* race used in the study; thus it should be re-classified as an all-stage resistance gene. The segregation frequency of genotypes carrying *Yr48* showed a strong distortion, possibly due to random drift or selection in gametes and/or zygotes. In addition, the genotype of nearby markers for this gene also showed distorted distributions, confirming the YR phenotypic results obtained in seedling.

### Interactions between Detected QTL

Small gene interaction between *QLr.cim-2DS* and *QLr.cim-3DC* may explain why the 3DC QTL did not display any significant interactions when combined with other QTL against LR in Avocet/Quaiu#3 population (Basnet et al., 2014). For YR, the effect of the *QYr.ucw-3BS* was larger in the presence than in the absence of *Yr48*, resulting in a highly significant interaction that explained a large proportion of the variation. Lowe et al. (2011b) also reported a highly significant interaction (*P* < 0.0001) between these two QTL with similar effects. No significant interactions were detected between *QYr.cim-3DC* and other QTL, suggesting that the YR resistance QTL on 3DC had an additive effect.

### Application

UC1110, a high yielding spring wheat line developed by the UC Davis breeding program, provides intermediate resistance to LR and YR in both Mexican and U.S. rust environments. PI610750 remained highly resistant to both rusts in the two countries. We obtained some transgressive RILs combining all of the detected YR resistance QTL, such as RILs 8, 26, 28, 119, and 254, with DS lower than 1% across Mexican and U.S. rust environments, whereas RILs 26 and 184 carry two LR resistance QTL resulting in DS of approximately 2%. We used two RILs (43 and 170) carrying the single resistance QTL 2DS and crossed them with RIL 180 (no resistance QTL) to develop single gene mapping populations for fine mapping and further characterization of this locus. The closely linked molecular markers to *QLr.cim-2DS* (within 0.5–2.3 cM), *QYr.ucw-3BS* (within 0.2 cM), and *Yr48* (within 0.6–0.7 cM) can be immediately used in wheat breeding programs to improve rust resistance via marker-assisted selection.

## Author Contributions

CL phenotyped the population, did analysis, and wrote the manuscript. IH and JD genotyped the population with DArT and SSR markers. SH-F, BB, MR, JH-E, and RS phenotyped the population at both seedling and adult plant stages.

## Conflict of Interest Statement

The authors declare that the research was conducted in the absence of any commercial or financial relationships that could be construed as a potential conflict of interest.

## Footnotes

1 https://wheat.pw.usda.gov/GG2/index.shtml
