# Supplementary material for: Characterization and Mapping of Leaf Rust and Stripe Rust Resistance Loci in Hexaploid Wheat Lines UC1110 and PI610750 under Mexican Environments
Source: Front Plant Sci. 2017 Aug 21;8:1450. doi: 10.3389/fpls.2017.01450 (PMC5573434; doi:10.3389/fpls.2017.01450)
Supplement: Supplementary file 1 [file Table_1.DOCX]

Table S1 Linkage map of *LrP* (*Lr26*) on wheat chromosome 1B

| Chromosome | Position | Markers |
| --- | --- | --- |
| 1B | 0.0 | *LrP* (*Lr26*) |
| 1B | 20.3 | wPt-1363 |
| 1B | 23.2 | barc8,rPt-2040,tPt-1772,tPt-5080,wPt-0170,wPt-0320,wPt-0328,wPt-0359,wPt-0655,wPt-0664,wPt-0725,wPt-0729,wPt-0831,wPt-1025,wPt-1116,wPt-1139,wPt-1176,wPt-1238,wPt-1251,wPt-1328,wPt-1346,wPt-1521,wPt-1568,wPt-1613,wPt-1717,wPt-1911,wPt-1912,wPt-1997,wPt-2019,wPt-2052,wPt-2057,wPt-2075,wPt-2261,wPt-2395,wPt-2540,wPt-2577,wPt-2614,wPt-2725,wPt-2762,wPt-2859,wPt-3177,wPt-3282,wPt-3315,wPt-3411,wPt-3587,wPt-3787,wPt-3824,wPt-3948,wPt-4107,wPt-4200,wPt-4306,wPt-4325,wPt-4326,wPt-4339,wPt-4434,wPt-4655,wPt-4786,wPt-5065,wPt-5067,wPt-5325,wPt-5435,wPt-5745,wPt-5765,wPt-5793,wPt-5798,wPt-5800,wPt-5801,wPt-5899,wPt-6078,wPt-6093,wPt-6117,wPt-6287,wPt-6370,wPt-6434,wPt-6442,wPt-6719,wPt-6777,wPt-6833,wPt-7094,wPt-7138,wPt-7359,wPt-7460,wPt-7529,wPt-7708,wPt-7833,wPt-7905,wPt-8177,wPt-8227,wPt-8261,wPt-8267,wPt-8287,wPt-8338,wPt-8532,wPt-8616,wPt-8949,wPt-8986,wPt-8996,wPt-9125,wPt-9283,wPt-9462,wPt-9472,wPt-9508,wPt-9522,wPt-9524,wPt-9528,wPt-9562,wPt-9605,wPt-9631,wPt-9776,wPt-9883,wPt-9903,wPt-9915,wPt-9975,wPt-9977,[rPt-9564,tPt-0325,tPt-6015,wPt-0014,wPt-0595,wPt-0734,wPt-1500,wPt-4343,wPt-4422,wPt-4652,wPt-5164,wPt-5312,wPt-6229,wPt-6427,wPt-6608,wPt-6831,wPt-7593,wPt-8776,wPt-8930,wPt-10703];barc187;rPt-0018,rPt-0079,rPt-1767,rPt-4523,rPt-5341,rPt-7959,rPt-8739,tPt-1051,tPt-2076,tPt-5515,tPt-5755,tPt-7918,tPt-9585,wPt-1781,wPt-7652,wPt-10960,[rPt-4471,rPt-9564,tPt-0325,tPt-6015,wPt-0014,wPt-0595,wPt-1500,wPt-4652,wPt-6229,wPt-6427,wPt-6608,wPt-6831,wPt-7593,wPt-8776,wPt-8930];wms374;rPt-1217,rPt-2869,rPt-3642,rPt-8894,tPt-0734,tPt-2240,tPt-2326,tPt-2550,tPt-3696,tPt-4566,tPt-7214,tPt-8754,[rPt-4471,rPt-9564,tPt-0136,tPt-1586];[rPt-9564,tPt-0136,tPt-1586,wPt-5164];wPt-0974;wPt-2575,[wPt-0734];wPt-3274,wPt-5279,wPt-9490,[wPt-4343,wPt-4422,wPt-5312,wPt-10703];wPt-3852,[wPt-0734];wPt-7259;wPt-10259 |
| 1B | 26.7 | gwm24 |
| 1B | 29.5 | wPt-1248 |
| 1B | 31.0 | wPt-5485 |
| 1B | 34.8 | wPt-8279 |
| 1B | 36.4 | cfd48-1B |
| 1B | 37.4 | wPt-8111 |
| 1B | 37.5 | wPt-0705 |
| 1B | 57.6 | tPt-7980,wPt-7160 |
| 1B | 60.8 | wPt-2257 |
| 1B | 64.7 | wPt-5217 |
| 1B | 66.4 | barc188 |
| 1B | 75.4 | wPt-10603 |
| 1B | 79.7 | rPt-9074,wPt-0944,wPt-2526,wPt-3475,wPt-4129,wPt-4532 |
| 1B | 80.8 | wPt-4688 |
| 1B | 87.3 | wPt-6619,wPt-8832 |
| 1B | 105.0 | wPt-4651 |
| 1B | 109.0 | wPt-1973,wPt-3950,wPt-6142 |
| 1B | 109.3 | wPt-9028 |
| 1B | 113.9 | barc80 |

Table S2. χ2 analysis of distorted molecular markers that are closely linked to *Yr48* on chromosome 5AL.

| Markers | No. of RILs with positive allele | No. of RILs with negative allele | No. of missing RILs | Expected No. of RILs with positive allele (1:1) | Expected No. of RILs with negative allele (1:1) | *P* value |
| --- | --- | --- | --- | --- | --- | --- |
| wPt-1903 | 117 | 57 | 12 | 87 | 87 | 5.4007E-06 |
| wPt-1038 | 122 | 63 | 1 | 92.5 | 92.5 | 1.4394E-05 |
| gwm595 | 123 | 63 | 0 | 93 | 93 | 1.0854E-05 |
| wmc524 | 124 | 62 | 0 | 93 | 93 | 5.4659E-06 |
| wmc727 | 120 | 57 | 9 | 88.5 | 88.5 | 2.1866E-06 |
| gwm410 | 125 | 57 | 4 | 91 | 91 | 4.6433E-07 |
| *Yr48* | 125 | 57 | 4 | 91 | 91 | 4.6433E-07 |
| wPt-9800 | 125 | 57 | 4 | 91 | 91 | 4.6433E-07 |
